# The Effectiveness of Mobile Applications in Improving Nursing Students’ Knowledge Related to Pressure Injury Prevention

**DOI:** 10.3390/healthcare12131264

**Published:** 2024-06-26

**Authors:** Mo`ath Nayef Alkhazali, Burcu Totur Dikmen, Nurhan Bayraktar

**Affiliations:** 1 Department of Surgical Nursing, Faculty of Nursing, Near East University, TRNC, Mersin 10, Nicosia 99138, Turkey; burcu.toturdikmen@neu.edu.tr; 2 School of Nursing, European University of Lefke, TRNC, Mersin 10, Lefke 99010, Turkey; 3 Department of Nursing, Faculty of Health Sciences, Atlılım University, Ankara 06830, Turkey; nurhan.bayraktar@atilim.edu.tr

**Keywords:** phone application, pressure injury, traditional method, nursing students

## Abstract

The study’s main goal was to compare the effects of a mobile application versus traditional methods of teaching on nursing students’ acquisition of knowledge about pressure injury prevention. In addition, a secondary aim was to discover nursing students’ viewpoints related to the advantages and disadvantages of using mobile applications as an educational method. A randomized controlled study design was implemented during November and December of 2023 in a nursing faculty with 60 undergraduate students in their second nursing year. A total of 30 students were assigned to the mobile application group, while the other 30 students were assigned to the traditional lecture group. The study was executed in three stages: pre-test, educational intervention, and post-test. The results of the study during the pre-test showed that there were no statistically significant differences in the mean scores of pressure injury themes between the two groups. However, the post-test scores for all PI themes were higher in the mobile application group compared to the traditional lecture group. Furthermore, five advantages of the mobile application were highlighted by students: “improvement of students’ knowledge and skills”, “self-confidence”, “stress reduction”, “enhancement of competence”, and “stimulation of learning motivation”. This study demonstrated the effectiveness of the mobile application method in enhancing nursing students’ knowledge and prophylaxis of pressure injury. Therefore, the mobile application method is recommended as an innovative approach to teaching.

## 1. Introduction

Recent developments in intelligent technology have opened up new avenues for innovation in teaching methods, especially within nursing and medical education. Utilizing online platforms, along with interactive learning methods like audience response systems, has proven to be successful in past studies focused on nursing education [1]. As the global population structure evolves, with an increasing aging population and higher prevalence of comorbidities, pressure injuries (PIs) have become a significant public health concern [2]. Various information exists regarding the frequency and occurrence of PI. Figures concerning hospitalized patients in Europe indicate a prevalence ranging from 6 to 28.2% [3,4]. Li Z et al., in a recent study, found that PIs affect approximately 12.8% of adults hospitalized worldwide, with an incidence rate of 5.4 per 10,000 patient days. [5]. Nurses hold a critical responsibility in averting PIs by delivering attentive nursing care to patients. Their role in preventive practices for PIs encompasses evaluating patients at risk of developing such injuries [6], which involves activities like applying wound dressing pads, administering massages and repositioning, and promoting overall health [7]. Nursing education plays an important role in preventing PIs. Previous research indicated that nurses possess restricted understanding regarding PI development [8] and preventative measures [9,10,11]. Research conducted previously revealed that nurses who regularly cared for patients with PIs and those who had training in PI management [11,12,13] demonstrated an enhanced understanding of the prevention and treatment of PIs. Consequently, the advancement of novel, engaging, and evidence-based training methods is presently a significant focus within clinical discourse.

Today, with advancements in mobile technologies, mobile phones are increasingly being utilized as educational tools in nursing education [14]. The mobile applications enable students to engage in learning, collaboration, and idea-sharing. Consequently, educational institutions must enhance their curriculum practices, emphasizing innovative teaching techniques and approaches as a matter of utmost importance [15]. Research on the integration of mobile applications in nursing education suggests that they contribute to the enhancement of students’ knowledge and skills [16]. Additionally, the utilization of educational mobile applications has been associated with improvements in students’ skills, satisfaction, and overall competence [17] while also potentially mitigating stress levels among them [18]. Mobile apps enable access to information, offer enjoyable learning experiences, and afford flexibility in terms of time and location for learning [19,20]. The usage of mobile devices in education underscores a shift from traditional educator-centered teaching to a more learner-centered educational approach [21].

Teaching through traditional lecture methods typically prioritizes exam performance over the quality of the depth of interactions and participation among students and educators [22]. While the traditional teaching approach remains prevalent in nursing schools, nursing students frequently encounter challenges in effectively grasping nursing content, actively listening, writing, synthesizing information, and translating acquired knowledge into clinical practice [23]. Sobirova and Karimova (2021) showed that traditional educational methods, which rely on repetition and memorization, hinder the development of critical thinking, problem-solving, and decision-making skills in students [24]. Therefore, it is crucial to devise educational methodologies that evolve in tandem with advancements and address the challenges encountered by undergraduate students. The number of studies comparing the mobile application teaching method with traditional methods related to nursing students has increased in recent years. These studies’ results have proven that the group using the mobile application teaching method had greater mean scores compared with traditional lecture methods [25,26,27]. Other studies that compared mobile applications with traditional lectures have reported that the use of mobile applications plays a key role in improving nurses’ skills, knowledge, increased confidence, and learning attitudes [28,29].

Recent studies emphasized the importance of using mobile technologies in education. Studies conducted to assess the importance of quality metrics in enhancing the usability of mobile learning systems during the COVID-19 pandemic. These studies stated that the key factors influencing learners’ satisfaction with mobile learning are service quality, information quality, and system quality [30,31]. A crucial element of mobile-based learning is its capacity to enhance critical thinking skills in students, which, in turn, promotes self-directed learning [32]. In their study, Ashiq et al. (2023) concluded that traditional learning often poses challenges for students in accessing books and learning resources due to the limitations of physical libraries and textbooks. In contrast, mobile-based learning offers convenient access to a wide range of learning resources via apps and the Internet, allowing students to download e-books, learning materials, and videos online [33]. Mobile-based learning has become a solution to the limitations of traditional educational methods. It provides a more engaging, accessible, and personalized approach to education, fitting well with the evolving demands of the digital age [34]. Farsangi et al. (2023) found that the mobile app-based cultural care training program positively impacted the cultural competence and humility of undergraduate nursing students [35]. Also, Shanmugapriya et al. 2023 concluded that Nursing students demonstrated positive acceptance and behavior toward using smartphones [36].

More studies are warranted despite the number of studies that have been conducted previously about using mobile application teaching methods in nursing education to confirm the results of these studies. As of now, no studies have been conducted in Cyprus on the integration of mobile applications in nursing education. Introducing innovative strategies for teaching PI topics can enhance nursing students’ understanding of PI prevention measures. Assessing the efficacy of a mobile application approach in preventing PIs could further facilitate the advancement of innovative methods for evidence-based nursing education. The study’s main goal was to compare the effects of a mobile application versus traditional methods of teaching on nursing students’ acquisition of knowledge about PI prevention. Additionally, the secondary aim was to discover nursing students’ viewpoints on the advantages and disadvantages of using mobile applications as an educational method. The research hypothesis is as follows: Nursing students who undergo the mobile application PI educational program will possess enhanced general knowledge and heightened awareness of the risk factors and prevention of pressure injuries compared to their counterparts in the traditional teaching group.

## 2. Materials and Methods

### 2.1. Study Design

The study was implemented using a randomized controlled design.

### 2.2. Study Sitting

The study was implemented during November and December of 2023 in a faculty of nursing from Northern Cyprus. The nursing curriculum mainly contains six nursing courses: Fundamental of Nursing, Medical-Surgical Nursing, Child Health and Disease Nursing, Obstetrical and Gynecological Nursing and Mental Health and Psychiatric Nursing. PI information is primarily covered in the Fundamental course, but it’s also referenced in other courses. Typically, conventional methods like PowerPoint lectures and in-class discussions are utilized for educational purposes.

### 2.3. Selection of Sample

The study was composed of 60 out of 100 undergraduate nursing students in their second year. Students were randomly assigned equally into two groups through the computerized randomization methods to ensure the homogeneity guaranteed for both groups. The pre-test results were analyzed during randomization. The sample consisted of the experimental and the control groups (30 students in each group). The sample size was determined using G-power analysis and based on a previous recent study [37]. Given a level of significance of alpha = 0.05, statistical power level of 0.8, and a medium effect size of (η2 = 0.06), the minimum required sample size is 60 in total, with 30 participants in each group being sufficient for a significant MANCOVA analysis.

### 2.4. Tools of Study

#### 2.4.1. Evaluation Form of PI Knowledge

The PI Knowledge Evaluation Form was used as a data collection tool in this study. The form was developed and validated by Beeckman et al. in 2010 [38]. A 26-item instrument was developed, reflecting 6 themes expressing the most relevant aspects of PI prevention. The content validity was excellent (CVI = 0.78–1.00). The overall internal consistency reliability (Cronbach’s A) was 0.77. The 1-week test-retest intraclass correlation coefficient (stability) was 0.88. The 1-week test-retest intraclass correlation coefficient (stability) was 0.88 [38]. This evaluation has also been used in previous studies in Australia [39]. Before applying the evaluation form, three specialists in the field of adult health nursing reviewed and approved it. The evaluation form was divided into two sections. The first section included demographic characteristics of students and four questions regarding age, gender, training department, and PI course. The second section consists of a knowledge assessment of PI, 26 multiple choice items, and three alternative responses reflecting six themes expressing the most relevant aspects of PI prevention: (1) etiology and development; (2) classification and observation; (3) nutrition; (4) risk assessment; (5) reduction in the magnitude of pressure and shearing; and (6) reduction in the duration of pressure and shearing.

#### 2.4.2. Students’ Opinions Evaluation Form

After finishing the post-evaluation test, participants were engaged in face-to-face interviews. Two open-ended questions regarding the advantages and disadvantages of the phone application. These questions were formulated as follows: “What are the advantages of utilizing the phone application method?” and “What are the disadvantages associated with the phone application method?”

#### 2.4.3. Mobile Application

The mobile application was funded by the researcher and developed by computer and information technology programmers. This application utilizes instructional videos, articles, and brochures to educate users about PIs. The home screen contained 7 icons. The first icon contained a 4–5 min video explaining the Integumentary System. The second contained two short videos: the first one, 1–2 min, explained the definition and risk factors of PI, while the second one, 3–4 min, explained the causes and development of PI. The third icon contained a 5–6 min video explaining the risk assessment of PI according to the Braden scale. The fourth icon contained a 6–7 min video explaining the stages of PI. The fifth icon contained information on the prevention of PI, and it had two sub-icons: the first one contained a 2–3 min video explaining the dressing and treatment of PI, and the second one explained the management of PI. The management icon also contained four sub-icons: the first one contained a 2–3 min video explaining the general management of PI, the second had a 1–2 min segment discussing nutrition, the third one had a 3–4 min video talking about repositioning and mobility, and the fourth icon contained a checklist discussing standard PI prevention protocols. The sixth icon contained two icons: the first one had a brief article, and the second one had a brochure explaining PI. The seventh icon contained contact information for researchers, such as phone number, email, and address. The duration of the educational activity ranged between 30–50 min. Prior to utilize the mobile application, three specialists in the adult health nursing field reviewed its contents and gave their approval for utilizing.

#### 2.4.4. Contents of Education

The educational material comprised three sections providing an overview of PIs and the PI prevention concepts:

- First part: Basic knowledge regarding PI and stages
- Second part: Risk factors of PI
- Third part: Evidence-based practices for PI prevention, including basic, physical, and pharmacological prophylaxis.

#### 2.4.5. Implementation

The study was carried out during November and December of 2023. To promote awareness, posters were used to announce the study, providing information about its purpose. Subsequently, researchers organized a meeting to elucidate the study’s objectives and the concept of mobile application learning. Following this, the study proceeded in three phases: pretest, educational intervention, and posttest for both groups. The timing of the study was carefully chosen to avoid interfering with the students’ midterm and final exams. Furthermore, participants voluntarily enrolled in the study without any form of compensation. They retained the right to withdraw at any point, with the assurance that their grades and future courses would not be affected by the study’s outcomes.

#### 2.4.6. Experimental Group

##### Pre-Phase

The pre-evaluation test was held, and all 30 students attended. The second part of the PI knowledge evaluation form was used. The exam took place face-to-face in the classroom, lasting one hour.

##### Intra-Phase

The students received an online link to download the mobile application. This application utilizes instructional videos and articles to educate users. The home screen contains icons for each category of PI, such as definition and causes, risk factors, risk assessment, stages, management, and prophylaxis. The duration of the educational activity ranged between 30–50 min.

##### Post-Phase

The post-evaluation test was held two weeks after the intra-phase. The same pre-evaluation test was repeated, and the students were asked to complete the second part of the PI knowledge evaluation form once again. Moreover, those in the Mobile application group were asked to respond to two open-ended questions regarding the pros and cons of mobile applications.

#### 2.4.7. Control Group

##### Pre-Phase

The pre-evaluation test related to the PI knowledge evaluation form was applied in the classroom. The timing, exam, and class were all identical to those in the experimental group, except that they were scheduled on a different day of the week.

##### Intra-Phase

The lecturer utilized conventional teaching techniques, which involved using PowerPoint presentations and distributing and handing out printed for the topic. In the class’ conclusion, students were provided with PowerPoints and/or printouts for review outside of class. No additional class activities or interventions were offered. The lecture lasted 2 h, including time for discussion.

##### Post-Phase

The same pre-evaluation test regarding knowledge of PI was administered again in the classroom.

### 2.5. Ethics

The study underwent assessment and approval by the institutional review board (IRB) under the reference number (2023\70\785), following the guidelines outlined in the Helsinki Declaration. Before the study commenced, written informed consent was obtained from all participating students. Students were reassured that their study participation would not affect their grades.

### 2.6. Statistical Analysis

The exam papers were securely collected in a locked locker located within the researcher’s office. SPSS V.28. was used to analyze the data, and numeric codes were utilized to input the data. A Kolmogorov–Smirnov normality assessment test was conducted to ascertain whether the data exhibited a normal distribution. Descriptive statistics, including frequencies and percentages, were utilized to analyze participants’ characteristics. Parametric statistical tests, specifically independent-sample *t*-tests, were employed to assess differences between study groups, with a chosen significance level of *p* < 0.05. To calculate the mean, each question was assigned a score of “one” for a correct answer and “zero” for an incorrect answer. Qualitative content analysis and frequency were employed to investigate both the pros and cons of utilizing the mobile application method, where themes pertaining to these aspects were identified.

## 3. Results

The findings indicated that the mean age of students in the traditional lecture group was 21.8 ± 1.3 years, while in the mobile application (intervention) group, it was 21.1 ± 1.1 years. Nearly all students had previous education in PI. No statistically significant distinctions in descriptive characteristics were observed between the study groups (*p* > 0.05). Consequently, the mobile application and traditional education groups were confirmed to be homogeneous (Table 1).

In the pre-test, there was no statistically significant difference observed in the mean scores of PI themes between the mobile application and traditional learning groups. The independent *t*-test comparing the mean scores of PI themes between the mobile application and traditional learning groups revealed a statistically significant difference. The post-test scores for all PI themes were higher in the mobile application group compared to the traditional lecture group (*p* < 0.05). Additionally, the paired *t*-test conducted for the mobile application group and traditional group comparing pre- and post-test mean scores demonstrated that all of the PI themes’ scores had improved statistically significantly for both groups (*p* < 0.05) (Table 2).

The strengths and weaknesses of mobile applications revealed that the majority of students (85%) highlighted the advantages, while 50% acknowledged the disadvantages (Table 3). Participants’ responses identified five categories related to the advantages of mobile applications: “improvement of students’ knowledge and skills”, “boost in self-confidence”, “stress reduction”, “enhancement of competence” and “stimulation of learning motivation”. On the other hand, the disadvantages of mobile applications were categorized into three groups: “time demands”, “technological requirements” and “cost implications”.

## 4. Discussion

The aim of this study was to evaluate how the use of a mobile application affects the knowledge and skills about PIs nursing students. Smartphones and other technological devices have become the initial and final points of interaction for humans [14]. Utilizing technology in education adds an element of enjoyment and excitement to the learning process, spanning various fields, including nursing education [40]. Chuang et al., (2022) demonstrated that e-book applications offer individuals the flexibility to manage their learning schedule and environment. Additionally, hands-on observation of procedural skills offers trainees feedback on techniques, enhancing the effectiveness of learning and the quality of PI care [41].

In this study, the main findings revealed no significant statistical variances in the average scores of PI categories between the two cohorts during the initial assessment. However, students instructed through the mobile application method exhibited higher mean scores across all PI categories compared to those taught through traditional methods. This trend persisted in the subsequent assessment, with the mobile application group maintaining higher mean scores in all PI categories compared to the traditional teaching group. These findings substantiate the study’s hypothesis that nursing students undergoing the mobile application PI educational program would possess broader knowledge and heightened awareness of PI risk factors and prevention compared to those in the traditional learning cohort, thus demonstrating the efficacy of employing mobile applications as a teaching tool.

Recent studies that showed using the mobile application teaching method has positive effects on the improvement of nursing students’ knowledge and skills [25,41,42]. The scores showed there are no significant statistical differences between the traditional lecture group and mobile application group in all of the PI themes during the pre-test, which indicates that basic knowledge is comparable. On the contrary, the scores showed a significant improvement in all PI themes in the mobile application group, and that is congruity with the study [29]. This is also what was reflected in a previous study conducted by Chuang et al. (2022), which confirms the effectiveness of e-book application teaching methods in improving nursing students’ information and awareness regarding PI prevention, and this confirms the validity of the hypothesis that nursing students who use mobile phone applications will possess a broader comprehension of PI general knowledge and they will also have an increased awareness of the risk factors and methods for preventing PIs [41].

In our study, the main results on the effectiveness of using the mobile application teaching method in enhancing nursing students’ knowledge and prevention regarding PI demonstrated. There is no significant differences in pre-tests between the two groups. This indicates that both groups started with a similar level of knowledge regarding pressure injury prevention. However, in the post-test, the group that utilized the mobile application for learning exhibited higher mean scores across all themes compared with the traditional lecture group. These results demonstrate the effectiveness of the mobile application method in educating nursing students about PI prevention and confirm the hypothesis of the study, which posited that “Compared to the traditional teaching group, nursing students who use the mobile application for PI prevention education applications they will possess a broader comprehension of PI general knowledge, and they will also have an increased awareness of the risk factors and preventative measures pertaining to PIs”. Major et al. (2021) and Chen et al. (2021) who concluded in their studies that mobile learning yields a positive impact on clinical nursing education for nursing students when compared to traditional methods [17,43].

Recent studies have underscored the significant positive impacts that innovative educational methods, such as mobile applications, have on nursing students’ learning outcomes. Our results align with the findings of Ghazanchaei et al. 2019 who found that e-learning significantly improved nurses’ knowledge of venous thromboembolism, and Khalid Al-Mugheed et al. 2021, who reported that the flipped classroom approach demonstrated positive outcomes in various domains related to venous thromboembolism (VTE) among nursing students, particularly in terms of knowledge acquisition, risk assessment, and understanding prophylaxis measures [44,45,46]. Studies indicated that using digital learning platforms for healthcare education has become more widespread and efficient. This confirms the effectiveness of this approach because it focuses on the learner and provides flexible access to information and activities online. Thus, mobile applications contribute positively to improving students’ knowledge and the quality of education [47].

On the other hand, the study conducted in 2021 by Khaled Al-mugeed et al. showed that there is no significant statistical differences in all of the mean scores according to the traditional lecture group compared with the flipped classroom group [45]. These results are consistent with our study, which showed that there is no significant statistical differences in all of the mean scores according to the traditional lecture group compared with the mobile application group.

A randomized experimental study with pre- and post-tests conducted in Taiwan involving 100 nursing students utilized a mobile app for clinical care learning. The results showed that the experimental group had significantly higher knowledge scores and greater satisfaction levels compared to the control group [48]. In addition, similar findings were observed in a controlled experimental study conducted in Turkey with 122 nursing students. This study indicated that the post-test results of the experimental group, which used an app for injection practices, showed a significant positive effect on knowledge levels (*p* < 0.05) [49]. A quasi-experimental study implemented in Brazil showed that the mobile application was deemed and led to a notable increase in nursing students’ knowledge, thus proving suitable for its intended purpose [50]. Coelho et al. (2021) concluded that the utilization of the application for therapeutic communication enhanced the knowledge of nursing students compared to the traditional teaching method [51]. Another study demonstrated that nursing students utilize mobile devices as an educational tool and found them effective in enhancing their knowledge and acquiring clinical skills [52]. Niromand et al. (2024) concluded that mobile-based learning is emerging as a significant educational approach with profound implications for healthcare education and the improvement of patient care quality. The widespread incorporation of mobile phones into the educational framework provides a flexible teaching paradigm, thus nurturing the potential for continuous lifelong learning [53]. All of the findings in these studies are compatible with our findings.

Moreover, the advantages and disadvantages of mobile applications provide qualitative feedback about implementing mobile learning applications in nursing education. The identified advantages included in our study were improvements in knowledge and skills, enhanced self-confidence, and stimulation of learning motivation, highlighting the multifaceted benefits of m-learning in nursing education. Conversely, the noted disadvantages—such as time demands, technological requirements, and cost implications—highlight areas for improvement and the need for institutions to provide adequate support and resources to maximize the effectiveness of these tools, and this is consistent with the results shown by studies [17,45].

### Limitations of Study

This study has several limitations. Since our study was conducted at a single nursing faculty, its findings cannot be generalized to broader populations or settings. This study focuses on knowledge of PI prevention more than skills. The limited number of included studies prevented the classification of different time points for outcome assessment, which could have influenced the results. The study solely examined the influence of certain aspects of mobile learning on academic performance, perceived satisfaction, and perceived usefulness. Consequently, future research exploring additional characteristics is suggested.

## 5. Conclusions

The present study demonstrates the effectiveness of the mobile application method in enhancing nursing students’ knowledge of PI. Compared to traditional lectures, mobile applications emerged as a more credible and efficient teaching tool. Moreover, the study elucidates both the advantages and disadvantages associated with mobile application use. It can be inferred that these findings hold significant implications for nursing education. The mobile application method exhibits considerable potential in preparing students for healthcare environments by fostering knowledge application, flexibility, and critical thinking skills. It is, therefore, recommended as an innovative and student-centered approach to teaching. Additionally, policymakers and educators can utilize these results to formulate strategies aimed at enhancing nursing education. However, further research with larger sample sizes and integration of this method into the nursing curriculum is warranted to fully explore its efficacy.

## Figures and Tables

**Table 1:** Descriptive characteristics of students (60 students).

Characteristics	Traditional Group		Intervention Group		*p* Value
	N	%	N	%	
Gender					
Male	21	70%	12	40%	0.15 ^a^
Female	9	30%	18	60%	
Training Department					
Medical	13	43.3%	11	36.7%	0.13 ^a^
Surgical	17	56.7%	19	63.3%	
Previous PI education					
Yes	28	93.3%	23	76.7%	0.2 ^b^
No	2	6.7%	7	23.3%	
	Mean	Sd	Mean	Sd	
Age					
	21.8	1.3	21.1	1.1	0.17 ^c^

Abbreviations: PI, Pressure Injury; ^a^ Fisher’s exact test; ^b^ X^2^; ^c^ The independent sample test.

**Table 2:** Comparison of PI means score of the traditional lecture and the mobile application group.

PI Theme	N of Items	Group	Pre-Test	Post-Test	*p* Value ^a^
			Mean Score ± SD	Mean Score ± SD	
Etiology and Development	5	Traditional lecture	2.2 ± 1.2	2.8 ± 1.2	0.11
		Mobile application	2.5 ± 1.3	3.9 ± 0.8	<0.001
		*p* value ^b^	0.12	<0.002	
Classification and observation	5	Traditional lecture	1 ± 0.8	1.2 ± 0.7	0.16
		Mobile application	1.1 ± 0.7	1.5 ± 0.7	<0.001
		*p* value ^b^	0.21	<0.004	
Risk Assessment	2	Traditional lecture	0.4 ± 0.5	0.6 ± 0.5	0.17
		Mobile application	1.1 ± 0.7	1.5 ± 0.7	<0.001
		*p* value ^b^	0.15	<0.001	
Pressure Injuries and nutrition	1	Traditional lecture	0.4 ± 0.5	0.6 ± 0.5	0.17
		Mobile application	0.5 ± 0.5	0.8 ± 0.4	<0.013
		*p* value ^b^	0.15	<0.001	
Preventative measures to reduce the amount of pressure/shear	7	Traditional lecture	3.5 ± 1.5	4.2 ± 1.4	0.14
		Mobile application	3.8 ± 1.6	5.6 ± 1.3	<0.001
		*p* value ^b^	0.18	<0.001	
Preventative measures to reduce the duration of pressure/shear	5	Traditional lecture	2.7 ± 1.1	3.1 ± 0.9	0.19
		Mobile application	3 ± 1.1	4.2 ± 0.7	0.001
		*p* value ^b^	0.13	<0.003	
Overall	25	Traditional lecture	12.3 ± 4.5	15.1 ± 3.7	0.15
		Mobile application	13.4 ± 4.4	20 ± 3.1	<0.001
		*p* value ^b^	0.24	<0.001	

Abbreviations: PI, Pressure Injury. ^a^ Paired *t*-test. ^b^ The independent sample test.

**Table 3:** Advantages and disadvantages of using mobile applications.

Advantage Items	N%	Disadvantage Items	N%
Improvement of students’ knowledge and skills	90%	Time demands	30%
Boost in self-confidence	85%	technological requirements	50%
stress reduction	82%	cost implications	70%
enhancement of competence	90%		
stimulation of learning motivation	85%		
total	85%		50%

## Data Availability

DATA based on request.
